# Clinical and histological characterization of oral pemphigus lesions in patients with skin diseases: a cross sectional study from Sudan

**DOI:** 10.1186/1472-6831-13-66

**Published:** 2013-11-21

**Authors:** Nada M Suliman, Anne N Åstrøm, Raouf W Ali, Hussein Salman, Anne C Johannessen

**Affiliations:** 1 Department of Clinical Medicine, The Gade Laboratory for Pathology, Faculty of Medicine and Dentistry, University of Bergen, Bergen, Norway; 2 Department of Clinical Dentistry, Faculty of Medicine and Dentistry, University of Bergen, Bergen, Norway; 3 Faculty of Dentistry, University of Science and Technology, Umdurman, Sudan; 4 Dermatology Clinic, Khartoum Teaching Hospital, Khartoum, Sudan; 5 Haukeland University Hospital, Bergen, Norway

**Keywords:** Oral pemphigus, Skin disease, Histology, Immunohistochemistry, Sudan

## Abstract

**Background:**

Pemphigus is a rare group of life-threatening mucocutaneous autoimmune blistering diseases. Frequently, oral lesions precede the cutaneous ones. This study aimed to describe clinical and histological features of oral pemphigus lesions in patients with skin disease has been canceled aged 18 years and above, attending outpatient’s facility of Khartoum Teaching Hospital - Dermatology Clinic, Sudan. In addition, the study aimed to assess the diagnostic significance of routine histolopathology along with immunohistochemical (IHC) examination of formalin-fixed, paraffin-embedded biopsy specimens in patients with oral pemphigus.

**Methods:**

A cross-sectional hospital-based study was conducted from October 2008 to January 2009. A total of 588 patients with confirmed skin has been canceled disease diagnosis completed an oral examination and a personal interview. Clinical evaluations supported with histopathology were the methods of diagnosis. IHC was used to confirm the diagnosis. Location, size, and pain of oral lesions were used to measure the oral disease activity.

**Results:**

Twenty-one patients were diagnosed with pemphigus vulgaris (PV), 19 of them (mean age: 43.0; range: 20–72 yrs) presented with oral manifestations. Pemphigus foliaceus was diagnosed in one patient. In PV, female: male ratio was 1.1:1.0. Buccal mucosa was the most commonly affected site. Exclusive oral lesions were detected in 14.2% (3/21). In patients who experienced both skin and oral lesion during their life time, 50.0% (9/18) had oral mucosa as the initial site of involvement, 33.3% (6/18) had skin as the primary site, and simultaneous involvement of both skin and oral mucosa was reported by 5.5% (1/18). Two patients did not provide information regarding the initial site of involvement. Oral lesion activity score was higher in those who reported to live outside Khartoum state, were outdoor workers, had lower education and belonged to Central and Western tribes compared with their counterparts. Histologically, all tissues except one had suprabasal cleft and acantholytic cells. IHC revealed IgG and C3 intercellularly in the epithelium.

**Conclusions:**

PV was the predominating subtype of pemphigus in this study. The majority of patients with PV presented with oral lesions. Clinical and histological pictures of oral PV are in good agreement with the literature. IHC confirmed all diagnoses of PV.

## Background

Pemphigus is a group of chronic inflammatory autoimmune bullous diseases. Although rare, they are potentially life-threatening diseases that are associated with high morbidity and mortality, if not properly treated [1,2]. The disease is associated with immunoglobulin (Ig) G and complement factor (C) 3 antibodies against intercellular adhesion structural components in the epithelium [3]. The immune reaction eventually breaks down the adhesion components and leads to epithelial cell detachment, which is clinically seen as intraepithelial blisters, erosions or ulcers in the skin and mucous membranes [4]. The underlying cause and activating mechanism that initiates the immune response is unidentified. However, both genetic and environmental factors have been postulated to play a role in the pathogenesis of pemphigus [5]. In this context, social habits like use of traditional cosmetics and smoking have been implicated [6-8].

Pemphigus has several subtypes, of which three have been associated with oral mucosal involvement; pemphigus vulgaris (PV), pemphigus foliaceus (PF), and paraneoplastic pemphigus [9]. The first two subtypes are differing with respect to the localization of intraepithelial blisters. In PV, the blisters are located suprabasally, while in PF they are more superficially located. Paraneoplastic pemphigus, although uncommon, is associated with internal malignant neoplasia [10].

Oral lesions present as vesicles or bullae that quickly break, leaving painful erosions or ulcers with irregular borders; they most often affect buccal mucosa and gingivae and heal slowly, without scarring. In PV, the oral lesions are reported as the initial sign of the disease in 50% of patients, yet these oral lesions have the greatest resistance to efficient treatment.

PV is the most predominant type of pemphigus, affects middle-aged adults without gender predilection [9,11-16] and has an incidence varying from 0.76 to 32 per million inhabitants per year [17-19]. While PV is a prevailing diagnosis in the Mediterranean region, South Asia and in the Jewish population [20,21], it is a rare disease in Northern Europe, USA, South Africa and northern region of Africa [6,18,19,22-24]. Reports from Mali and South Africa have shown that PV is rare in the black ethnicity [22,24].

Diagnosis of pemphigus is based on careful correlation of disease history and clinical findings with histopathologic characteristics. Direct immunofluorescence (DIF) on sections from a fresh frozen biopsy or indirect immunofluorescence (IIF) performed on patient’s serum are important for verifying the diagnosis [25]. However, in situations where IF is difficult to perform, immunohistochemistry (IHC) on formalin-fixed tissue samples may be an alternative test to confirm the diagnosis [26].

A study conducted in a dermatology clinic of Khartoum Teaching Hospital (KTH) in Sudan in 1998, focusing mainly on skin lesions, showed that PV was the dominant variant of pemphigus, constituting 88% of all cases diagnosed [27]. According to that study, oral mucosa was the second most common site of the lesion to occur after the trunk. The highest frequency of PV was found in the third decade of life [27]. Another study conducted in the same clinic in 2008, revealed a prevalence of oral PV of 2.8% among skin diseased outpatient attendees [28]. This study also showed that the frequency of oral PV among patients with skin disease with any oral mucosal lesions was 4.8%. In both studies, clinical information and conventional histological examination of biopsies using haematoxylin and eosin (H&E) staining were the only methods for diagnosing skin lesions.

Sudan is a large country with a multi-cultural multi-ethnic society. The prevailing ethnicities are Arabic and African, with hundreds of tribal divisions. The epidemiologic profile is typical of Sub-Saharan African countries; malaria, infectious diseases, hypertension, diabetes mellitus, and nutrition disorders are among the prominent diseases treated in the health units of Sudan [29]. On the basis of these considerations and due to the scarce information available regarding pemphigus in sub-Saharan African populations, the present study, presenting a further analysis of the data conducted in 2008 [28], aimed to describe clinical presentation of patients with oral pemphigus attending the dermatologic clinic of KTH. Given the fact that conventional histology was the only diagnostic tool in public hospitals in Sudan, the study also evaluated the diagnostic significance of combining this technique with IHC analysis of the formalin-fixed, paraffin-embedded oral biopsy specimens.

## Methods

### Sampling procedure

A cross sectional hospital based study was carried out focusing on patients aged ≥18 years with mucocutaneous diseases, attending an outpatient dermatologic clinic at KTH from October 2008 to January 2009. KTH is the largest national hospital in Sudan, located in Khartoum, the capital city. It is an open public and referral hospital, receiving patients from all the states of the country. For the present study, a minimum sample size of 500 patients was calculated based on an assumed prevalence of oral mucosal lesions (OML) in patients with skin diseases of 5%, a confidence interval of 95%, and an absolute precision of 0.02 [30]. All patients (n = 4235) attending the outpatient facility during the survey period were invited to participate in the study. A total of 1540 subjects (36.4%) initially accepted to participate. Fear of taking biopsy for asymptomatic lesions and time consuming examinations (oral examination, interview, and biopsy when needed) were the main reasons for not volunteering to participate. Of those who initially accepted to participate, 588 (588/1540, 38.1%) patients were finally included in the study.

Confidentiality of the patients was maintained, participants were informed about their oral conditions, and health education was provided. Those who needed dental services were referred to the clinics of the Faculty of Dentistry, University of Science and Technology (UST), Umdurman, for further investigation and management. Written informed consent or finger print (illiterates) for participation and publication of the study was obtained from patients or their parents/guardians. The research conformed to the Helsinki Declaration, and ethical clearance and approval letters were obtained from the participating institutions’ committees in Sudan (UST and KTH, Department of Dermatology) and Norway (The Regional Committee for Medical Research Ethics of Western Norway).

### Socio-demographic characteristics and clinical examination

A structured questionnaire was administered by two trained dentists in face to face interviews. *Socio-demographic characteristics* were measured in terms of gender, age, tribe, occupation, marital status, place of residence and oral habits. Participants were also asked about history of PV among first-degree relatives (parents, grandparents, siblings, children, and grandchildren). Medical condition and treatment were assessed according to the following conditions: heart diseases, hypertension, asthma, diabetes, liver diseases, hepatitis /jaundice, anaemia, bleeding disorders, kidney diseases, rheumatoid arthritis, allergy, cancer, epilepsy, stomach ulcer, intestinal disorders, respiratory disorders, pregnancy, psychiatric treatment, radiotherapy and chemotherapy. Furthermore, the patients were asked if their medical condition was diagnosed by a specialist and if they were under medication.

An expert dermatologist (HS) evaluated the patient’s skin diseases based on history of the disease and clinical findings, and the diagnosis was subsequently confirmed by histological examination when it was considered necessary. Details of involved sites at presentation and clinical course of the lesions were registered.

Systematic comprehensive extra-oral and intra-oral clinical examinations based on visual inspection and palpation, following the World Health Organization (WHO) criteria for field surveys [31], were carried out by a dentist (NMS) who received a training in diagnosis of OML before the data collection (The Gade Institute, Section for Pathology, and Department of Clinical Dentistry, Section for Oral Surgery and Oral Medicine, University of Bergen, Norway). An OML was defined as any abnormal change or any swelling in the oral mucosal surface. Diagnostic criteria for OML were based on Axéll’s criteria and those defined in former studies and reviews [31-33]. The oral clinical examination and additional information with respect to OML and oral habits have been reported elsewhere [28]. Data on location, size, clinical presentation of the oral lesion (vesicle, erosion/ulcer) and clinical course were recorded. Skin lesions and oral lesions were encountered during the survey and were photographed using a digital camera (Canon EOS 400D). Final diagnoses of all biopsies were given by an expert oral pathologist (ACJ).

### Assessment of clinical oral lesions’ activity

To assess the clinical severity of the oral lesions, an oral lesion activity score (OLAS) was constructed. The score was based on three components. Firstly, clinical extension of the OML was assessed. A modified system based on an established protocol [34] was used to register the extension of an oral lesion at10 anatomical locations; upper lip, lower lip, gingival mucosa, unilateral buccal mucosa, bilateral buccal mucosa, tongue, floor of the mouth, hard palate, soft palate and oropharynx. Each location was assessed as 0 = no lesion, 1 = presence of lesion, resulting in a total score ranging from 0 to 10. Secondly, size of the lesion was determined according to the largest diameter of a lesion at any location present at examination and scored as; 1 < 1 cm, 2 ≥ 1 cm. Thirdly, severity of symptoms was evaluated by asking patients to describe any pain associated with eating and drinking and was reported as: 0 = no pain, 1 = mild to moderate pain, and 2 = severe pain. Based on a former report [35], the OLAS for each patient was constructed as the sum of objective score (location, size) and subjective score (pain), ranging from 1 to 14, and reported in terms of means.

### Assessment of oral tissue biopsy

Oral tissue biopsies were taken from the periphery of the lesions. The tissue was fixed in formalin and embedded in paraffin. Sections were stained with hematoxylin and eosin (H&E) and examined using light microscope. To evaluate inflammation, number of inflammatory cells (mononuclear and polymorphonuclear cells) in the superficial parts of the connective tissue adjacent to the tip of the epithelial rete ridges, were counted in 6 random fields (one field = 250 μm^2^) per section using an ocular grid and high power magnification (40 ×). The inflammatory cells were counted 3 times per each field, and results were expressed as a mean per specimen (mean ± SD/1500 μm^2^). The variation of degree of inflammation between specimens was evaluated.

### Procedure for immunohistochemistry on formalin-fixed, paraffin-embedded oral tissue

IHC for IgG and C3 was performed on formalin-fixed, paraffin-embedded oral mucosal specimens from 11 patients. Sections, 4 μm-thick, were cut on a Leica RM2155 microtome and mounted on glass slides (Super Frost Plus, Gerhard Menzel Gmbh, Germany) and heated at 56°C overnight. The sections were deparaffinized in xylene and rehydrated in alcohol. For C3c, sections were incubated in target retrieval solution (pH6, S1699, DAKO, Glostrup, Denmark), microwaved for 15 minutes after the buffer had come to a boil, then let to cool down on bench and thereafter washed slightly under running tap water for 5 minutes. Primary anti-human C3c polyclonal rabbit compliment (A0062, DAKO) at 1:15000 dilutions was incubated for 30 minutes at room temperature. For IgG, sections were incubated in epitope retrieval solution (proteinase type XXIV bacterial, Sigma P 8038 37) for 10 minutes at 37°C. Primary antibody polyclonal rabbit anti-human IgG (A 0423, DAKO) at 1:60000 dilutions were incubated for 60 minutes. Endogenous peroxidase activity was blocked by 0.03% hydrogen peroxide (H_2_O_2_) (S2023, DAKO) for 7 minutes. Detection was performed using peroxidase labeled polymer conjugated to goat anti-rabbit/mouse immunoglobulins (K5007, Envision + ®, DAKO) for 30 minutes. Between each of the above steps, sections were washed with tris-buffered saline with Tween (TBST, pH 7.6, S3306, DAKO) for 10 minutes. Reaction was then visualized using 3, 3′- diaminobenzidine (DAB) (K5007, DAKO). The sections were thereafter counterstained with hematoxylin (S3301, DAKO), dehydrated and mounted with a non-aqueous mounting medium (Eukitt, O.Kindler GmbH & Co., Freiburg, Germany).

### Statistical analysis

Descriptive statistical analysis was done using PASW Statistics version 18.0 (SPSS Inc., Chicago, USA).

## Results

A total of 588 outpatients participated in the study. Out of those participants, there were 22 patients with pemphigus, where PV was the most frequent disease (95.4%, 21/22) followed by PF (4.5%, 1/22). Fourteen patients (63.6%, 14/22) were already diagnosed, coming with new active lesions, while 7 patients (31.8%, 7/22) were newly diagnosed cases. In one patient there was no information about disease history. Of the 588 patients, 359 had at least one type of OML, while oral PV was registered in 19 patients.

### Demographic features of patients with oral PV

Of the 19 patients diagnosed with oral PV (mean age 43.0, range 20–72 yrs), 10 were females (mean age, 35.8 yrs) and 9 were males (mean age, 38.3 yrs). None of the females were pregnant. As shown in Table 1, the majority of the patients were <50 yrs (68.4%), low education (84.2%), married (77.8%), had outdoor jobs (52.6%), and were residing outside the Khartoum state (57.9%). Patients who reported Western tribes were 47.4% (9/19) compared to 21% (4/19) from Northern tribes, 26.3% (5/19) from Central tribes and only one reported Southern tribes. Totals of 11.1%, 21.1%, and 10.5% confirmed use of toombak, smoking and use of alcohol, respectively. These habits were exclusively reported by males.

**Table 1:** **Socio-demographic distribution of study participants with oral PV according to gender and means of oral lesions activity scores** (**OLAS)**

	**Female**	**Male**	**OLAS**
	**n (%)**	**n (%)**	**n (Mean ± SD)**
**Age**			
< 50 years	8 (80.0)	5 (55.6)	11 (8.7 ± 3.5)
≥ 50 years	2 (20.0)	4 (44.4)	5 (8.6 ± 2.3)
**Education**			
Low education (illiterate + primary)	9 (90.0)	7 (77.8)	13 (9.3 ± 2.8)
High education	1 (10.0)	2 (22.2)	3 (6.0 ± 3.0)
**Marital status**			
Unmarried	2 (20.0)	2 (25.0)	4 (8.0 ± 3.7)
Married	8 (80.0)	6 (75.0)	11 (8.7 ± 3.1)
**Occupation**			
Indoor job (professional, skilled labour and unemployment)	6 (60.0)	3 (33.3)	7 (6.7 ± 3.2)
Outdoor job (farmer, animal breeder, street seller and builder)	4 (40.0)	6 (66.7)	9 (10.2 ± 2.0)
**Tribal distribution**			
Northern region	2 (20.0)	2 (22.2)	4 (5.7 ± 2.0)
Southern region	1 (10.0)	0	1 (7.0)
Western region	5 (50.0)	4 (44.4)	8 (9.6 ± 3.0)
Central region	2 (20.0)	3 (33.3)	3 (10.6 ± 2.5)
**Residence during last 5 years**			
Khartoum state	5 (50.0)	3 (33.3)	6 (6.1 ± 2.3)
Out of Khartoum state	5 (50.0)	6 (66.7)	10 (10.2 ± 2.5)
**Habits**			
Toombak user	0	2 (25.0)	1 (6.0)
Non-user	10 (100)	6 (75.0)	14 (8.8 ± 3.2)
**Smoker**	0	4 (44.4)	4 (8.0 ± 0.8)
Non-smoker	10 (100)	5 (55.6)	12 (8.9 ± 3.5)
Alcohol user	0	2 (22.2)	2 (7.5 ± 0.7)
Non-user	10 (100)	7 (77.8)	14 (8.8 ± 3.3)

The total number in the different categories did not add to 19 owing to missing values.

### History and aggravating factors

Negative family history (first degree- relatives) was reported by all patients. The systemic conditions reported by the patients included hypertension (5 patients), intestinal problems (4 patients), and allergy, anaemia, arthritis, and diabetes each was reported by 3 patients. Moreover, peptic ulcer, hepatitis, thyroid and liver diseases were reported by one patient each. No neoplastic diseases were recorded. Aggravating factors like eating spicy food, stress and smoking were reported by 3 patients. Concerning medications, one patient had taken penicillin and another one co-trimoxazole before eruption of the disease. All patients were scheduled to be treated with systemic steroids.

### Clinical presentation of oral lesions of PV

**Figure 1:**
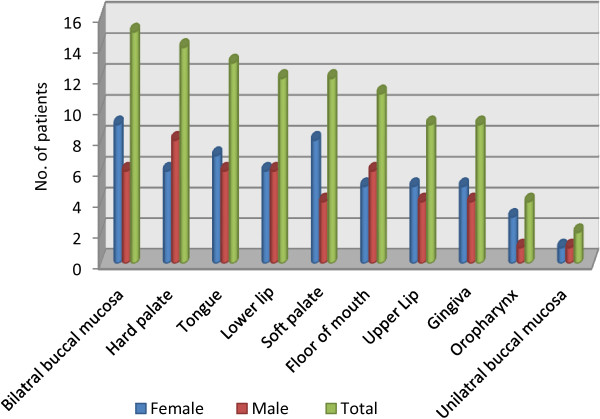
Distribution of oral manifestations in patients with pemphigus vulgaris.

At time of examination, 76.1% (16/21) of the patients with PV had both oral and skin lesions. Exclusively oral lesions were observed in three females 14.2% (3/21), and a former history of skin lesions was reported by two of them. In patients who experienced both skin and oral lesions during their life time, 50.0% (9/18) had oral mucosa as the initial site of involvement, 33.3% (6/18) had skin as the primary site, and simultaneous involvement of both skin and oral mucosa was reported by 5.5% (1/18). Two patients did not provide information regarding the initial site of involvement. In addition to oral lesions, extremities and trunk were the most common cutaneous sites involved followed by scalp, genitalia and eyes. As shown in Figure 1, bilateral buccal mucosa was the most commonly affected site followed by hard palate. The sites least affected were oropharynx and unilateral buccal mucosa. The clinically predominant oral lesions were mucosal erosions and ulcers. Vesicles were evident in one patient only.

Oral lesions which were > 1 cm in diameter were registered in 52.6% (10/19), and those which were ≤ 1 cm in diameter were registered in 47.4% (9/19) of patients. Pain was reported as severe by 43.8% (7/16), moderate by 37.5% (6/16) and no pain by 16.7% (3/18) of patients. Missing information was noted in each category of pain description. With respect to the OLAS total scores, scores 3 and 4 were registered in one patient each (6.3%), while 6, 7, 8, 9, 11, 12 and 13 total scores were recorded in two patients each (12.5%). The total mean of the OLAS was 8.27 (range 3–13).

As shown in Table 1, the mean of the OLAS was high with those who resided out of Khartoum state and with outdoor workers (10.2) compared with those living in Khartoum state (6.1) and indoor workers (6.7). Also, it was high with lower education (9.3) compared to higher education (6.0), and with those who reported Central (10.6) and Western (9.6) tribes compared to other tribes from Northern and Southern parts of Sudan.

### Microscopic examination

Eleven out of 16 patients with oral lesions agreed to have a biopsy taken. The histological characteristics were comparable in all tissue specimens. Ten out of eleven biopsies were covered by non-keratinized epithelium. Few inflammatory cells (lymphocytes and neutrophils) were present in the superficial epithelial layer of 5 biopsies. No candida hyphae could be demonstrated by PAS staining. Nearly all biopsies demonstrated spongiosis in the lower spinous cell layers, in addition to presence of neutrophils and lymphocytes in 6 biopsies. Apoptotic cells were seen in the spinous layer of 3 specimens.

**Figure 2:**
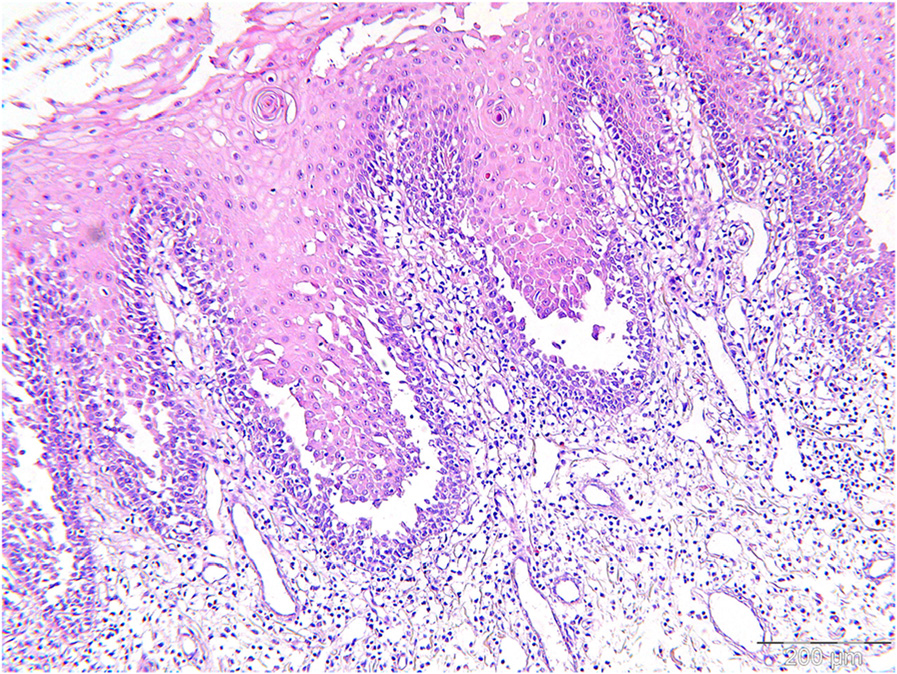
**Histology of oral mucosa of pemphigus vulgaris shows acantholysis in the lower spinous cell layers.** Basal layer cells are attached to the connective tissue and suprabasal cleft are seen at the tips of the epithelial rete ridges (scale = 200 μm).

Suprabasal epithelial clefts were detected in 10 out of 11 biopsies, while one patient biopsy showed spongiosis only. However, in that patient, the diagnosis of PV was based on histopathology of a skin biopsy. In some sections, 1 to 2 layers of suprabasal keratinocytes were attached to basal cells forming part of floor of the cleft. In 6 biopsies, the clefts were especially seen on the tip of the epithelial rete ridges (Figure 2). The basal cells forming the floor of the cleft varied between areas in the same section as well as between sections, displaying complete loss of intercellular attachment (tombstones) or showing intact attachment, where all basal cells remaining attached to the basement membrane and lamina propria. Inside the cleft, partially and completely detached keratinocytes (acantholytic cells) from the basal and lower prickle cells layers were spotted as single cells or clusters. In addition, lymphocytes and neutrophils were the main inflammatory cells inside the cleft.

In all tissue sections, the superficial parts of the connective tissue were characterized by edema, small blood vessels, loose fiber arrangements and both interstitial and perivascular inflammatory infiltrates. Mononuclear cells were the principal inflammatory cells, and there were only few neutrophils and eosinophils. The total number of mononuclear cells varied across the 6 fields for each specimen (Table 2), from 151 cells (mean: 25.1 ± 4.2; range: 22–31 cells) to 407 cells (mean: 67.8 ± 10.2; range: 53–79 cells). Mononuclear cells in the specimens were not influenced by the level of the OLAS. Deeper in the connective tissue, mast cells and perivascular mononuclear cell infiltrates were seen in 7 specimens.

**Table 2:** Distribution of mononuclear cells in the superficial parts of the connective tissue adjacent to the tip of the epithelial rete ridges (6 random fields per section)

**Patients**	**Number of mononuclear cells**						**Sum (Mean ± SD)**	**Range**
	**Area 1**	**Area 2**	**Area 3**	**Area 4**	**Area 5**	**Area 6**		
1	68	26	32	66	38	85	315 (52,5 ± 23,7)	26–85
2	40	45	33	35	44	38	235 (39,1 ± 4,7)	33–45
3	30	22	22	31	22	24	151 (25,1 ± 4,2)	22–31
4	44	15	18	39	43	60	219 ( 36,5 ± 17,0)	15–60
5	47	28	19	13	16	48	171 (28,5 ± 15,5)	13–48
6	36	37	44	43	32	84	276 (46 ± 19,1)	32–84
7	48	28	49	42	46	45	258 (43 ± 7,7)	28–49
8	24	19	13	28	29	50	163 (27,1 ± 12,6)	13–50
9	53	79	77	73	60	65	407 (67,8 ± 10,2)	53–79
10	75	38	45	44	73	70	345 (57,5 ± 16,8)	38–75
11	70	50	50	24	47	60	301 (50,1 ± 15,3)	24–70

### Immunohistochemistry

**Figure 3:**
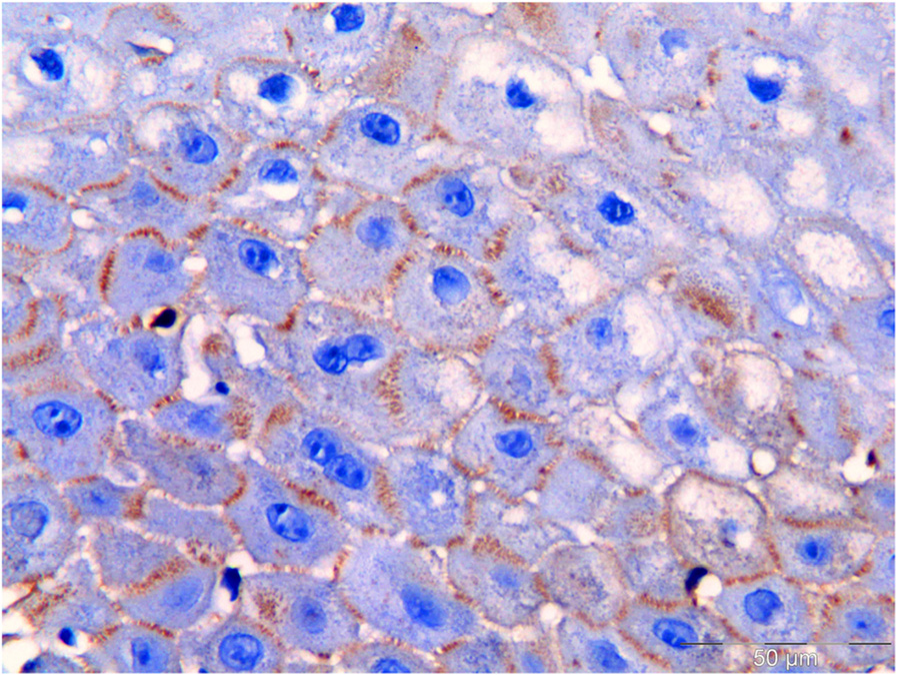
**Immunohistochemistry staining used to detect IgG in formalin-fixed, paraffin-embedded oral tissue biopsy from patients with pemphigus vulgaris.** IgG (brown colour) is seen in the intercellular junction of keratinocytes reliable with the location of desmoglein 3 (scale = 50 μm).

**Figure 4:**
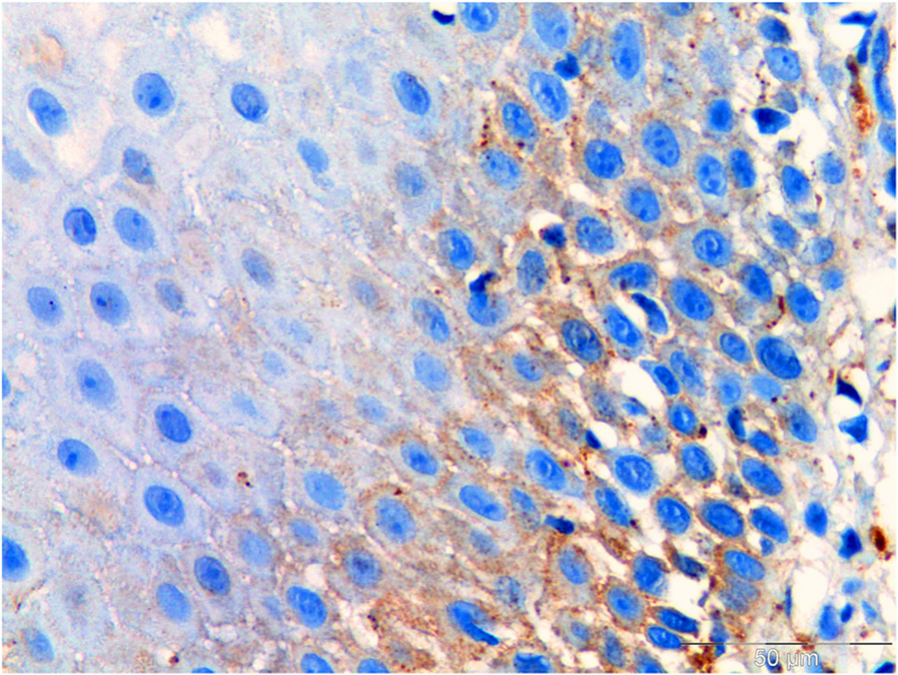
**Immunohistochemistry staining used to detect C3 in formalin-fixed, paraffin-embedded oral tissue biopsy from patients with pemphigus vulgaris.** C3 (brown colour) is seen in the intercellular junction of keratinocytes reliable with the location of desmoglein 3 (scale = 50 μm).

IgG and C3 were detected intercellularly in the epithelium of all specimens examined. The staining was strongest in the suprabasal layer of the epithelium (Figures 3 and 4).

## Discussion

This is the first study to report on the clinical characteristics of patients with oral pemphigus in Sudan, specifically in outpatients of a dermatology clinic in KTH. In this study, the prevailing variant of pemphigus was PV (95.4%), and oral mucosal involvement was present in 90.4% of the patients. An initial oral involvement was reported by 50% of those with both skin and oral lesions. The majority of the patients were in their fifth decade of life. Palate and buccal mucosa were the most common locations followed by tongue and lower lip. Based on the OLAS, the highest severity of the oral lesions was found in patients with low education, having outdoor jobs, from Central and Western tribes, living out of Khartoum state and being non-smokers. The histopathological pictures of all specimens were in agreement with the IHC findings. However, our findings should be interpreted with caution since several limitations were inherited in the study design. The cross –sectional hospital based design of this study and the small sample size of the study populations hindered statistical evaluation of the findings. In addition, the relatively short period for data collection and the potential effect of selection bias were considered to influence the results and limit generalization. While the KTH is one of the largest national referral hospitals in Sudan, other referral and private hospitals could also receive patients from other parts of Khartoum and the rest of the country. In spite of the limitations mentioned above, the study may be beneficial as a first step in studying a new issue and to generate hypotheses.

Pemphigus is primarily considered to be a dermatologic disease. The fact that PV commonly and initially affects the oral mucosa and then the skin [16], gives dentists a great opportunity to detect the disease at an early stage. The present study showed that 90.4% of the patients had oral mucosal lesions, which is in accordance with a previous report [36]. Moreover, a multicentre study by Brenner et al. [37], found varying prevalence of oral lesions in patients with PV; 66% in Bulgarian patients, 83% in Italian, and 92% in Israeli patients. Our result is higher than those reported by Ramirez et al. [38], who found a prevalence of 18% of oral lesions in PV patients examined in a dermatologic clinic in Mexico City.

Generally, PV has been reported to affect men and women equally [9]. Several studies registered the highest frequency in females [21,39-46], while a few studies have reported males’ dominance [41,47]. In general, estrogen (exogenous and endogenous) has been accused for the females’ predominance in autoimmune diseases [48]. Supporting the hypothesis, 80% of the females in the present study were in the premenopausal period (< 50 years old). A prospective case–control study among Tunisian females found that traditional cosmetics, commonly used after marriage (henna, kohl, souak), was associated with occurrence of pemphigus diseases in younger women [6]. In that respect, we found that 80% of women were married. Sudanese married women use on daily basis traditional home-made cosmetics like skin exfoliating scrub paste, oils, perfumes, henna, and smoke-baths, beside modern cosmetic skin whiteners, which are used among both married and single women.

Evidences from Western countries indicate that autoimmune diseases are increasing in frequency and show a female predominance [48,49]. Accordingly, some authors hypothesized that this could be, at least partially, attributable to new or modified patterns of exposure to chemicals, including environmental estrogens [48]. Many organochlorine pesticides are suspected to impair natural hormonal function in organisms by mimicking endogenous estrogen. The hypothesis proposes that the impact of pesticides and gardening materials on estrogen metabolism can trigger pemphigus. Our result showed that among outdoor workers, farming was the outdoor job most frequently reported. One may speculate that beside sun exposure, environmental estrogens may affect specific population with a susceptible genetic background.

Traditionally, PV tends to appear between the ages of 40 and 60 years [9]. In the present study the mean age was 43 years, close to findings from Thailand, Spain and Korea [15,42,50], but lower when compared with data given from countries like Romania, Germany and North America [51-53] and higher than other countries like Kuwait and Iran [14,54].

Educational achievement is connected with better employment and income, which in turn can affect health behaviors and access to health facilities and thus treatment in appropriate time [55]. That could partially explain the higher frequency of PV and the higher OLAS mean values among the low educated group as well as outdoor job workers in comparison to their counterpart. In addition, outdoor workers are likely to be exposed to sun light and UV radiation for a long time of the day. The band of the UV radiation has been suggested to induce pemphigus [56,57]. Also, heat might be necessary to liberate a sufficient amount of PV antigen from epithelium [58]. Moreover, mid latitude, subtropical and tropical countries have been suspected to have higher frequency of pemphigus than other countries. Thus, higher incidence was found in Mediterranean countries [20,59] compared to high latitude countries like Finland and North America [18,19]. An epidemiological study from Greece demonstrated that high temperatures and extreme sun exposure raise the relapse frequency of PV [59]. Another study from South Africa observed exacerbation of pemphigus during summer time [24]. In connection with that, Sudan is located between latitude 4 to 22 degrees north, characterized by an environment range from tropical climate in the south, to savannah and desert in the central and northern area where temperature normally exceeds 40°C, especially during summer [60,61]. Controversially, one study from Iran showed that higher rate of disease onset was in winter [54]. Another study from Tehran found that there was no significant difference of disease onset or recurrence among annual seasons, indicating that genetic and racial variations might play a more important role than climate differences in the pathogenesis of PV [62].

PV was the predominant variant of pemphigus in the present study (95.4%). This was in accordance with several previous studies [11-15], but opposing data from some African regions (Mali, South Africa, Tunisia and Libya) where PF was the dominant variant [22-24,63]. The data from Mali and South Africa proposed that PV is rarely seen in black African ethnicity. Sudan has two ethnic groups; Afro-Arab tribes and non-Arab African tribes, where Southern tribes belong to the latter one. In the present result, PV was registered in only one patient from Southern tribes of Sudan compared to 9 (47.4%) from Western tribes (Afro-Arab and non-Arab African tribes). Yet the disease was not registered in Sudanese Eastern tribes (non-Arab African tribes) which lead to unexplained results. Several heterogeneous factors have been implicated of inducing or triggering pemphigus in different ethnic populations including genetic factors, in particular Human Leukocyte Antigen (HLA) class II loci. That has been noted in Ashkenazi Jews, Iranian, Italian patients and also in patients of South Asian and Mediterranean origin [64,65]. In comparison, the disease seems to be rare in Northern Europe and USA. However, genetic factors alone is not enough to initiate the autoimmune reaction as demonstrated by a report of PV in only one of two monozygotic twins [66]. Our data showed an absence of PV in first degree relationship. This is supported by the paucity of familial PV reports in the literature as reviewed by Tetsuya et al. [67]. It is conceivable that exogenous factors might induce PV in genetically predisposed individuals.

Cigarette smoking has been a highly controversial topic with respect to effect on certain autoimmune diseases. Although it is considered one of the leading morbidity and mortality risk factors [68], some clinical evidence has supported its beneficial and protective effect on patients with pemphigus [69] and certain diseases such as ulcerative colitis and recurrent aphthous ulcers [70,71]. Thus, smoking history is an essential factor in pemphigus patients. A case control study by Brenner et al. [37], reported that risk for PV was lower in current and ex-smokers than for patients who had never smoked. It has been shown that short-term exposure to nicotine might control keratinocyte adhesion by increased motility, proliferation and lateral migration. That would increase re-epithelialization and rapid wound healing. On the other hand, opposite results were shown when patients were subjected to chronic or long-term exposure to nicotine [72,73]. The effect of chronic exposure to nicotine was shown to reduce cutaneous blood flow and inhibit and decrease fibroblast migration in wound healing and to induce wound infection [74,75]. Moreover, immunosuppressive effects in terms of reduction in immunoglobulins, helper/suppressor T-cell ratios, lymphocyte transformation and natural killer cell cytotoxicity could also delay the healing process. Nevertheless, varying outcomes are still recorded [76]. In association with that, the present study showed that smoking was exclusively reported by males and in only 21.1% (4/19) of the patients. Although non-smokers and smokers registered similar OLAS means, the latter were the less frequent.

In this study, patient’s medical history revealed some medications that were taken before the disease eruption such as penicillin, co-trimoxazole and anti-hypertensive drugs, indicating a possible association, which has also been suggested in other studies [5,77,78].

The present results showed that half of the patients with oral lesions had experienced the first lesion in the mouth followed by skin lesions. This is in accordance with a study performed in India (53.5%) [79]. The 14.2% (3/21) of patients with exclusively oral lesion found in our study is comparable to 16.5% from Iran [80], but in contrast to 86% found in Northern Greece [44]. Exclusively oral lesions tend to be a marker for less virulent disease and a better prognosis [81]. It is uncommon to clinically identify vesicles or bullae in the oral mucosa due to continuous mechanical forces that characterize the normal activity of the mouth. Beside the thin and fragile roof of the PV bullae, irregular erosions and ill-defined ulcers were the principal clinical features of oral PV. Our findings notified only one patient with bullae, contradicting a Brazilian study that found a rate of 75% of patients with clinically identifiable vesicles [82]. The distribution of oral lesions in our PV patients followed the international literature showing the most common sites were palate, buccal mucosa, tongue and lower lip [83]. Other studies found the gingiva to be the most commonly affected site [84,85]. Although gingival lesions are uncommon at the onset, they usually manifest as severe desquamative or erosive gingivitis in advanced stages of PV [86].

Based on histology, we were able to confirm the diagnosis in 10 out of 11 specimens. The microscopic examination was found to be comparable with the classic histology of PV as described in the literature. This result reinforces previous suggestions, that oral lesions of pemphigus preserve the histologic features of acantholysis more than in skin lesions, and they are less prone to bacterial infections than skin, where secondary infections may affect the definitive histologic pattern [87]. Oral ulcers were the main clinical characteristic features of the patients in this study. Therefore, to demonstrate the typical pathologic changes in PV, all specimens in the present study were taken from intact oral mucosa immediately adjacent to the lesions. Considering inflammatory infiltrates, the results showed that mononuclear cells were the principal inflammatory cells in all specimens, confirming the chronic nature of PV. Furthermore, evidences have shown that lymphocytes play a critical role in immune surveillance and activation in PV. Lymphocytes have been reported to produce specific cytokines that may be critical for the launch and continuation of the production of Dsg3-specific autoantibodies by B lymphocytes [88,89]. Our results showed moderate variations in the numbers and means of inflammatory cells from one specimen to another, as well as in the range of cells within one specimen. These results are in consistence with the literature [10].

Tzanck smear to detect acantholytic cells is used as a screening procedure and rapid test for diagnosing oral PV [90]. A recent report has shown that Candida smear using methylene blue was useful in detecting acantholytic cells in such cases [91]. Some studies found that the efficacy of H&E staining alone in diagnosing PV is probably greater than 90% and could be considered satisfactory [92,93]. However, ambiguous cases and treatment planning remain challenging. Equivocal cases come from a number of conditions that are expressed as oral vesicles or bullae that rapidly rupture and result in erosions or ulcers. Some are viral infections such as herpes simplex infection, while others are immunologic diseases like pemphigus, pemphigoid, lupus erythematosus and lichen planus. In such cases, DIF and IIF are important to discriminate between them, confirm diagnosis and plan proper treatment. Limitations of these techniques are the availability of blood serum or fresh frozen tissue that requires specific facilities to perform, and they do not always exist in all health services, especially in developing countries. It also is important to secure safe transport to laboratories using correct media that prevent autolysis of the tissue. In addition, it is costly and thus not affordable for all patients. Thus, pathologists in Sudan usually receive patients’ specimens that have been fixed in formalin or normal saline [94]. To overcome these limitations, a previous study applied DIF on formalin-fixed, paraffin-embedded tissue [95]. Although the technique was less sensitive than when using frozen tissue, the possibility of misclassification was low. Another study using immunoperoxidase staining technique on formalin-fixed, paraffin-embedded tissue from PV patients, proved the possibility of detecting immunoglobulin in the absence of microscopic features of the disease [96]. Our study demonstrates that IHC on formalin-fixed, paraffin- embedded tissue is a reliable method to confirm the diagnosis of PV. At the time of the present study, and specifically in public hospitals in Sudan, confirmation of the PV was based on clinical examination, anamnesis and conventional histopathology. Thus, IHC enhances the accuracy of diagnosing PV and can be used when only formalin-fixed, paraffin-embedded tissue is available for analysis.

## Conclusion

PV was the predominating subtype of pemphigus in this study. The majority of PV presented with oral lesions. The results of this study are in agreement with the previous studies with respect to the age, gender, oral lesions distribution and first presentation of PV. The aetiology of PV is uncertain, but several heterogeneous factors could implicate to its pathogenicity. IHC in formalin-fixed, paraffin-embedded oral tissue biopsy confirmed the diagnosis of PV. The current study shed light on the higher prevalence of oral PV among the study population, suggesting that great collaboration efforts between dermatologists and dentists would provide better treatment and avoid serious sequelae and death.

## Competing interests

The authors declare that they have no competing interests.

## Authors’ contributions

NMS was the main author who conceived and designed the study, collected data, performed statistical analysis and drafted the manuscript. ANÅ participated in the study design, statistical and epidemiological data analyses. RWA facilitated the field work. HS was the main dermatologist who examined and diagnosed all the patients. ACJ was the main-supervisor, participated and guided the study design and confirmed and approved all the diagnosis of oral lesions. All authors read and approved the final manuscript.

## Pre-publication history

The pre-publication history for this paper can be accessed here:

http://www.biomedcentral.com/1472-6831/13/66/prepub
